# Hepatoprotective Effect of the Aqueous Extract of *Simarouba amara* Aublet (Simaroubaceae) Stem Bark against Carbon Tetrachloride (CCl_4_)-Induced Hepatic Damage in Rats

**DOI:** 10.3390/molecules191117735

**Published:** 2014-10-31

**Authors:** Hélida M. L. Maranhão, Carlos F. B. Vasconcelos, Larissa A. Rolim, Pedro J. Rolim Neto, Jacinto da C. Silva Neto, Reginaldo C. da Silva Filho, Mariana P. Fernandes, João H. Costa-Silva, Alice V. Araújo, Almir G. Wanderley

**Affiliations:** 1 Department of Pharmaceutical Sciences, Universidade Federal de Pernambuco, Recife 50740-521, Brazil; 2 Department of Histology and Embriology, Universidade Federal de Pernambuco, Recife 50670-901, Brazil; 3 Department of Physical Education and Sport Science, Universidade Federal de Pernambuco, Vitória de Santo Antão 55608-680, Brazil; 4 Department of Nutrition, Universidade Federal de Pernambuco, Vitória de Santo Antão 55608-680, Brazil; 5 Department of Physiology and Pharmacology, Universidade Federal de Pernambuco, Recife 50760-901, Brazil

**Keywords:** Simaroubaceae, *Simarouba amara*, carbon tetrachloride, catechin, hepatoprotection, antioxidant

## Abstract

*Simarouba amara* stem bark decoction has been traditionally used in Brazil to treat malaria, inflammation, fever, abdominal pain, diarrhea, wounds and as a tonic. In this study, we investigate the hepatoprotective effects of the aqueous extract of *S. amara* stem bark (SAAE) on CCl_4_-induced hepatic damage in rats. SAAE was evaluated by high performance liquid chromatography. The animals were divided into six groups (n = 6/group). Groups I (vehicle—corn oil), II (control-CCl_4_), III, IV, V and VI were pretreated during 10 consecutive days, once a day p.o, with Legalon^®^ 50 mg/kg b.w, SAAE at doses 100, 250 and 500 mg/kg b.w, respectively. The hepatotoxicity was induced on 11th day with 2 mL/kg of 20% CCl_4_ solution. 24 h after injury, the blood samples were collected and their livers were removed to biochemical and immunohistochemical analyzes. The SAAE decreased the levels of liver markers and lipid peroxidation in all doses and increased the catalase levels at doses 250 and 500 mg/kg. Immunohistochemical results suggested hepatocyte proliferation in all doses. These results may be related to catechins present in SAAE. Thus, SAAE prevented the oxidative damage at the same time that increased regenerative and reparative capacities of the liver.

## 1. Introduction

In Brazil, the Simaroubaceae family is represented by Quassia and Picrolemma genera in the Amazon region, Castela and Picrasma in the south; Simaba and Simarouba in almost all Brazilian regions [1]. *Simarouba amara* Aublet, popularly known as “praíba”, “marupá” and “pau-paraíba”, is a large tree that reaches up to 40 m height and 0.5 to 0.9 m diameter [2]. The use of *Simarouba amara* has a long history in folk medicine of many countries. Ethnopharmacological data suggest the use of a cup of *S. amara* stem bark decoction, 2–3 times per day, to treat malaria, inflammation, fever, abdominal pain, diarrhea, wounds and as a tonic [3,4]. *Simarouba amara* pharmacological assays showed antimalarial action of its fruits [5], amoebicide and bactericidal activities (against *Shigella flexineri* and *Salmonella typhosa*) of its stem bark and its root bark showed moisturizing action in the human epidermis [3].

Liver disease refers to any disorder of the liver and includes the following conditions: steatosis or fatty deposits in the liver; fibrosis or scarring of the liver; hepatitis or inflammation of the liver; cirrhosis and liver cancer [6]. The oxidative stress is one of the main mechanisms involved in pathology and thus natural products are being investigated as a source of antioxidants to treat liver disease [7,8,9].

Phytochemical screening of *S. amara* showed alkaloids, triterpenes and quassinoids from *S. amara* stem bark [10]. Moreover, six new triterpenoids were isolated from the *S. amara* stem bark and two other compounds previously known (3-oxatirucalla-7,24-dien-23-ol and niloticin) [11]. Alkaloids and tannins also have been identified in the bark of this specie [12]. Although these metabolites are widely shown to present antioxidant properties, the hepatoprotective effect of *S. amara* has not been explored. Therefore, the present study investigated the possible hepatoprotective potential of the aqueous extract of *Simarouba amara* stem bark (SAAE) against CCl_4_-induced oxidative stress in liver of rats.

## 2. Results and Discussion

### 2.1. Chromatographic Analyzes

#### 2.1.1. Thin Layer Chromatography

Phytochemical analysis of the aqueous extract from *Simarouba amara* stem bark demonstrated the presence of hydrolyzable tannins (gallic and ellagic acids) and condensed tannins (proanthocyanidins and leucoanthocyanidins). Phenylpropanoids and cinnamic acid derivatives (cafeic acid) were also identified, which are phenolic compounds known to present antioxidant properts. Traces of saponins, steroids and quassinoids were recorded. These results allowed to select the patterns used in the high performance liquid chromatography.

#### 2.1.2. High Performance Liquid Chromatography

**Figure 1:**
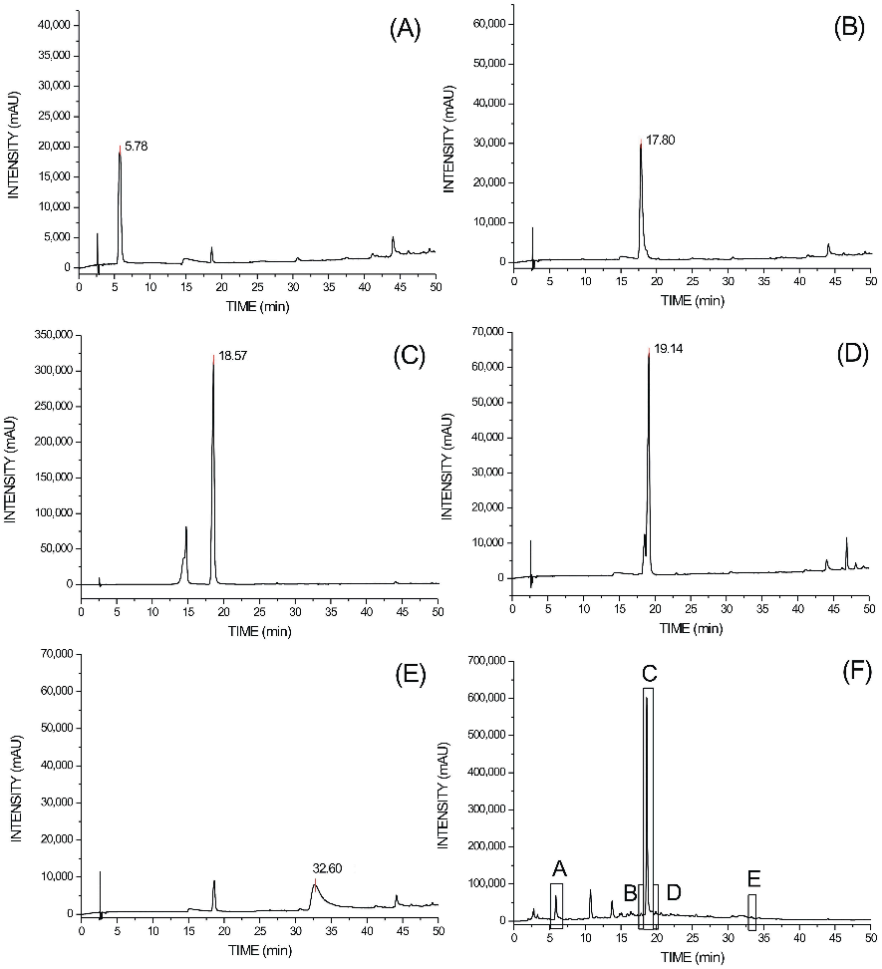
HPLC traces. Peaks: (**A**) gallic acid; (**B**) chlorogenic acid; (**C**) catechin; (**D**) epicatechin; (**E**) ellagic acid; (**F**) aqueous extract of *S. amara* stem bark (A: gallic acid; B: chlorogenic acid; C: catechin; D: epicatechin; E: ellagic acid).

It was observed the presence of chromatographic peaks consistent with the patterns. Patterns retention times were 5.78, 17.80, 18.57, 19.15 and 32.6 min for gallic acid, chlorogenic acid, catechin, epicatechin and ellagic acid, respectively. The retention times of the main metabolites present in the aqueous extract of *S. amara* stem brak were 5.76, 17.9, 18.57, 19.14 and 32.6 min (Figure 1), respectively.

The metabolites concentrations (x) present in the SAAE were obtained of the regression equation created by reference compounds, where Y is mean of the peak areas. The equations obtained were: gallic acid (Y = 7972.5 + 46676 x, R^2^: 0.9996); chlorogenic acid (Y = −14411 + 39623.6 x, R^2^: 0.9996); catechin (Y = 133758.92 + 325788.25 x, R^2^: 0.9998); epicatechin (Y = −32128.5 + 46676.2 x, R^2^: 0.9995) and ellagic acid (Y = 7972.5 + 46676.2 x, R^2^: 0.9996).

The concentrations of these compounds in the SAAE were16.47, 3.83, 349.96, 16.43 and 1.29 μg/mL, respectively. Linear regression analysis indicated linearity of the method. The HPLC analysis quantified the mainly metabolites observed in the preliminary phytochemical screening and the catechins were the major components quantified in the extractive solution (21.8%).

### 2.2. Evaluation of Lipid Peroxidation, Catalase and Superoxide Dismutase Levels

The administration of CCl_4_ to rats was shown to cause oxidative stress in the liver as can be seen by the augmented levels of malondialdehyde (MDA), which was prevented by pre-treatment with all doses of SAAE. We have not observed lower activities SOD and CAT induced by CCl_4_ in the control animals. However, there is a significant increase in the CAT levels of the SAAE treated animals at doses 250 and 500 mg/kg in relation to control group (Table 1).

**Table 1:** Effects of the aqueous extract of *Simarouba amara* stem bark (SAAE) on lipid peroxidation, catalase and superoxide dismutase levels in Wistar rats.

Groups	MDA (nmol/mg Protein)	CAT (mU/mg Protein)	SOD (U/mg Protein)
Control	5.94 ± 0.88	0.33 ± 0.03	11.96 ± 0.06
Vehicle	3.88 ± 0.44 ^a^	0.46 ± 0.04 ^b^	11.89 ± 0.17 ^b^
Legalon^®^	3.19 ± 0.36 ^a^	0.40 ± 0.02 ^b^	11.57 ± 0.62 ^b^
SAAE 100 + CCl_4_	3.42 ± 0.37 ^a^	0.45 ± 0.05 ^b^	12.22 ± 0.13 ^b^
SAAE 250 + CCl_4_	2.69 ± 0.42 ^a^	0.92 ± 0.12 ^a^	11.77 ± 0.21 ^b^
SAAE 500 + CCl_4_	3.56 ± 0.55 ^a^	0.83 ± 0.02 ^a^	12.17 ± 0.34 ^b^

The values represent mean ± S.E.M (n = at least 4 animals per group). ^a^ Statistically different from control (CCl_4_ group). ^b^ No statistical differece from control (CCl_4_ group). (ANOVA followed by Newman-Keuls, *p* < 0.05). MDA (ANOVA followed by Newman-Keuls, *p* < 0.05). MDA: Malondialdehyde; CAT: Catalase; SOD: Superoxide dismutase.

Carbon tetrachloride (CCl_4_)-induced hepatic toxicity is a tool used as an experimental method to study the hepatoprotective effect of natural products and drugs. The first step in CCl_4_ metabolism is a one-electron reduction catalyzed by cytochrome P450, producing •CCl_3_ [13]. The •CCl_3_ radical is highly reactive. In the presence of molecular oxygen, most of the •CCl_3_ radicals generated react to form the trichloromethylperoxyl free radical, •OOCCl_3_ [14]. Both radicals are capable of binding to proteins or lipids, leading to membrane lipid peroxidation and finally cell necrosis [15,16].

Lipid peroxide radicals, lipid hydroperoxides, and lipid breakdown products develop in this process, and each constitutes an active oxidizing agent. Consequently, cell membrane structures and intracellular organelle membrane structures are completely broken down and structural damage spreads [9]. An approach for the detection of hepatic injury involves measurement of lipid peroxides, such as malondialdehyde (MDA) [17]. The increase in MDA levels in the liver suggests enhanced peroxidation leading to tissue damage and failure of the antioxidant defense mechanisms to prevent the formation of excessive free radicals [18].

The antioxidant system is very critical for the detoxification of free radicals. Superoxide dismutase (SOD) diminishes the concentration of highly reactive superoxide radical (O_2_^−^) by converting it to H_2_O_2_, whereas catalase decomposes H_2_O_2_ and protect the tissues from highly reactive hydroxyl radicals (•OH^−^). These antioxidant enzymes are easily inactivated by lipid peroxides or reactive oxygen species, which results in decreased activities of these enzymes in CCl_4_ toxicity [8,19].

In the present study, we have not observed lower activities SOD and CAT induced by CCl_4_ in the control animals, probably due to organism adaptation mechanism against stress [20]. However, the treatments with the doses of 250 and 500 mg/kg SAAE have induced an increase in CAT levels, which may prevent the oxidative damage induced by CCl_4_.

### 2.3. Biochemical Parameters

The administration of CCl_4_ to rats resulted in marked elevation of serum enzymes ALT, AST, LDH and bilirubin. Membrane disintegration of hepatocytes with subsequent release AST, ALT and LDH, among others, is one of the consequences of CCl_4_-induced lipid peroxidation [21]. Treatment with SAAE in all doses reversed the increased levels of these enzymes and GGT levels were reduced only in the group treated with the highest dose (Table 2).

**Table 2:** Effects of the aqueous extract of *Simarouba amara* stem bark (SAAE) on biochemical parameters in Wistar rats.

Groups	AST (U/L)	ALT (U/L)	AF (U/L)	GGT (U/L)	TB (mg/dL)	DB (mg/dL)	IB (mg/dL)	LDH (U/L)
Control	2001.0 ± 7.4	2478 ± 6.5	125.6 ± 6.4	2.2 ± 0.6	0.42 ± 0.07	0.26 ± 0.08	0.13 ± 0.03	2560.0 ± 8.8
Vehicle	85.5 ± 4.8 ^a^	43.7 ± 2.3 ^a^	96.8 ± 5.5	0.2 ± 0.2 ^a^	0.10 ± 0.00 ^a^	0.08 ± 0.01	0.01 ± 0.01 ^a^	328.6 ± 3.6 ^a^
Legalon^®^	213.7 ± 8.7 ^a^	120.8 ± 5.0 ^a^	100.0 ± 8.3	1.5 ± 0.6	0.25 ± 0.15 ^a^	0.20 ± 0.10	0.05 ± 0.05 ^a^	421.5 ± 5.5 ^a^
SAAE 100 + CCl_4_	501.8 ± 9.3 ^a^	576.5 ± 6.3 ^a^	92.7 ± 8.7	0.8 ± 0.3	0.10 ± 0.00 ^a^	0.10 ± 0.00	0.00 ± 0.00 ^a^	599.5 ± 16.4 ^a^
SAAE 250 + CCl_4_	419.7 ± 7.7 ^a^	541.0 ± 14.2 ^a^	91.2 ± 7.5	0.8 ± 0.4	0.10 ± 0.00 ^a^	0.10 ± 0.00	0.00 ± 0.00 ^a^	462.8 ± 7.9 ^a^
SAAE 500 + CCl_4_	252.8 ± 5.4 ^a^	220.6 ± 6.9 ^a^	86.2 ± 6.5	0.3 ± 0.2 ^a^	0.12 ± 0.00 ^a^	0.10 ± 0.00	0.02 ± 0.00 ^a^	337.8 ± 2.6 ^a^

The values represent mean ± S.E.M (n = 6/group). ^a^ Statistically different from Control (CCl4 group) (ANOVA followed by Newman-Keuls, *p* < 0.05). AST: aspartate aminotransferase; ALT: alanine aminotransferase; AF: alkaline phosphatase, GGT: gamma—glutamyltranspeptidase; TB: total bilirubin; DB: direct bilirubin; IB: indirect bilirubin; LDH: lactate dehydrogenase.

Estimation of serum enzymes, such as aspartate and alanine aminotransferases and lactate dehydrogenase, is a useful quantitative marker of the extent and type of hepatocellular damage. Increases in these enzymes levels have been attributed to damage of the structural integrity of the liver, because these enzymes are released into the circulation after autolytic breakdown or cellular necrosis of the hepatocytes [22]. Thus, the treatment with SAAE reversed these parameters and avoided hepatocyte damage and loss of its functional integrity.

### 2.4. Proliferating Cell Nuclear Antigen (PCNA) Analysis

**Figure 2:**
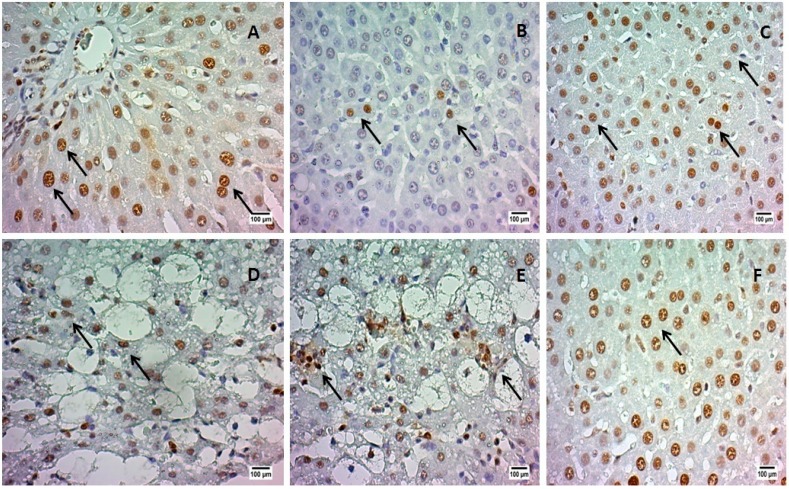
PCNA immunohistochemical analyzes of rats’ livers treated with aqueous extract of *Simarouba amara* stem bark (SAAE). (**A**) Vehicle (corn oil); (**B**) Control (CCl_4_); (**C**) Legalon^®^; (**D**) SAAE 100 mg/kg b.w.; (**E**) SAAE 250 mg/kg b.w.; (**F**) SAAE 500 mg/kg b.w. (500× increased). PCNA: positive nuclei (brown spots indicated by arrows).

This assay revealed a great number of proliferating cells in the liver of the animals treated with SAAE in all doses (intense immunoreactivity) when compared to control (Figure 2). The dose of 500 mg/kg of SAAE had an immunoreactivity similar to Legalon^®^ (over 75% of positive nuclei).

The treatment with SAAE stimulated the proliferation of the hepatocytes, as assessed by the proliferating cell nuclear antigen (PCNA) method. PCNA is a protein that is expressed in the G1 late phase and during S phase of the cellular cycle, being used as a marker of cell proliferation [23]. The greater PCNA reactivity in the animals treated with SAAE indicates that it avoids hepatocyte injury since it is able to reverse an established damage in the liver, through stimulation of the proliferation. It has been reported that tannic acid prevented DNA damage of the liver and kidney cells of adult chickens [24].

It has been shown that CCl_4_-induced liver injury is significantly decreased by the treatment with tannins, probably due to its antioxidant effect [25]. The antioxidant and free radical scavenging properties are the catechins’ most renowned biological actions. In this study, catechins, a type of condensed tannin, showed the highest concentration in the SAAE. Our hypothesis is that the catechins present in the SAAE were responsible for the antioxidant activity and consequently for the hepatoprotection presented by the SAAE.

## 3. Experimental Section

### 3.1. Chemical and Reagents

Catechin, epicatechin, gallic, ellagic and chlorogenic acids, carbon tetrachloride (CCl_4_), thiobarbituric acid (TBA), trichloroacetic acid (TCA), epinephrine and sodium carbonate were purchased from Sigma-Aldrich Chemicals Co. (St. Louis, MO, USA). Aspartate aminotransferase (AST), alanine aminotransferase (ALT), bilirubin, alkaline phosphatase (AF), γ-glutamyl-transpeptidase (GGT) and lactate dehydrogenase (LDH) were purchased from Boehringer Ingelheim^®^ (São Paulo, Brazil). Proliferating cell nuclear antigen (PCNA) was purchased from Abcam (Cambridge, MA, USA) and Legalon^®^ (active principle—silymarin) from (Nycomed Pharma, São Paulo, Brazil).

### 3.2. Material Plant

The stem barks of *Simarouba* *amara* Aublet were collected in São João, Pernambuco, Brazil (08°52'33"S and 36°22'01" The Gr) and identified at the Agronomic Institute of Pernambuco. A voucher specimen was deposited at the Dárdano de Andrade Lima Herbarium under number 85268.

### 3.3. Extract Preparation

The stem barks of *Simarouba* *amara* were collected in December 2012. The samples were shade dried for 48 h and then placed in a circulating air oven at a temperature of 45 ± 2 °C to stabilize the residual moisture. At the end, the barks were ground in a knives mill. The aqueous extract of *Simarouba amara* stem bark (SAAE) was obtained from the decoction of the powder (10:100 w/v) using distilled water as extractor solvent for a period of 10 min. The aqueous extract was concentrated in lyophilizer. The yield of *Simarouba amara* dried extract was 12.15 g per liter of the SAAE.

### 3.4. Chromatographic Analyzes

#### 3.4.1. Thin Layer Chromatography

The SAAE was evaluated for the presence of hydrolyzable tannins (gallic and ellagic acids), tannins condensed (catechins), flavonoids, saponins, coumarins, phenylpropanoids, cinnamic acid derivatives, alkaloids, triterpenes/steroids, monoterpenes, sesquiterpenes, quassinoids, iridoids and sugars. The phytochemical profile was assessed in silica gel chromatographic plates (Merck^®^ art. 105553, UV 250–366 nm) using mobile phases, reagents and appropriate standards [26].

#### 3.4.2. High Performance Liquid Chromatography

HPLC conditions: chromatographic analysis to quantify tannins and chlorogenic acid in the extractive solution were conducted in liquid chromatograph Shimadzu^®^ (UFLC, Kyoto, Japan) controlled by software LC Solution 1.0 and consisting of LC-20 AT pump, degasser DGU-20A5, Sil-20A autosampler and detector diode array (DAD) SPD-M20A. It was used Restek^®^ C18 column (250 mm × 4 mm, 5 μm) maintained at 30 °C. The standards and samples were eluted using a mobile phase gradient consisting of methanol (A) and 0.5% acetic acid (pH 3.0) (B). The conditions were: 0–50 min (10%–90% A and 90%–10% B), 50–55 min (90%–10% A and 10%–90% B), 55–60 min (10% A and 90% B). Flow rate of 0.8 mL/min and injection volume of 20 μL. The chromatograms were obtained at 290 nm.

Sample preparation: the SAAE was used in the concentration of 1.6 mg/mL. For patterns, it was prepared aqueous solution of catechin (0.16 mg/mL), gallic acid (0.16 mg/mL), ellagic acid (0.16 mg/mL), epicatechin (0.1 mg/mL) and methanolic solution of chlorogenic acid (0.05 mg/mL). The patterns concentrations were used to construct their analytical curves. All samples and patterns were filtered through membranes of 0.22 μm (Millipore^®^) and injected in triplicate.

### 3.5. Animals

Male Wistar rats (*Rattus norvegicus* var. *albinus*) (aged 2 months, weighing 250–280 g) were obtained from the Department of Physiology and Pharmacology at the Federal University of Pernambuco (UFPE), Pernambuco, Brazil. The animals were kept under standard environmental conditions (22 ± 2 °C; 12:12 h dark/light cycle). Water and industrialized dry food (Presence^®^, Purina, Paulínia, Brazil) were available *ad libitum*. On September 2012, the experimental protocol was approved by the Animal Experimentation Ethics Committee of UFPE (Process no. 026449), in accordance with the National Institute of Health Guidelines for the Care and Use of Laboratory Animals.

### 3.6. Treatment

The animals were randomly divided into six groups (n = 6/group). The liver injury was induced according to Simile *et al.* [27] with slight modifications. Groups I (vehicle—corn oil), II (control-CCl_4_), III, IV, V and VI were pretreated during 10 consecutive days, once a day p.o, with Legalon^®^ 50 mg/kg b.w (active principle—silymarin), SAAE at doses 100, 250 and 500 mg/kg b.w, respectively. The doses were fixed based on preliminary studies (data not shown). The hepatotoxicity was induced on 11th day by administering 2 mL/kg of CCl_4_ (20% diluted in vehicle). The animals fasted overnight with access to water *ad libitum* and 24 h after injury, the blood samples were collected by rectro-orbital puncture for biochemical analysis and the animals were euthanized with anesthetic depth using Nembutal^®^ until the dose of 140 mg/kg, i.p. and their livers were carefully removed.

### 3.7. Liver Analyzes

#### 3.7.1. Liver Homogenate

Homogenate of liver tissue was prepared in 50 mM Tris buffer containing 1 mM EDTA (pH 7.4), 1 mM sodium orthovanadate, 2 mM phenylmethanesulfonyl fluoride (PMSF) and centrifuged at 2500× *g* for 10 min at 4 °C. The supernatant was collected and used in the following experiments as described below. Concentration of protein in supernatant was estimated using crystalline bovine serum albumin (BSA) as standard [28].

#### 3.7.2. Lipid Peroxidation Assay

Oxidative stress was estimated by using the level of thiobarbituric acid-reactive substances (TBARS) [29]. In the TBA test reaction, malondialdehyde (MDA) or MDA-like substances react to produce a pink pigment with maximum absorption at 535 nm. Briefly, the reaction was developed by the sequential addition of sample (100 μL of homogenate), trichloroacetic acid 30% and Tris-HCl 10 mM (pH 7.4). The mixture was centrifuged at 2500× *g* for 10 min. After centrifugation, the supernatant was transfered to another tube and it was added TBA 0.8% (v/v). The mix was boiled in water-bath for 30 min. After cooling, the absorbance of the organic phase was read at 535 nm in a spectrophotometer. Results were expressed as nmol per mg of protein (nmol/mg protein).

#### 3.7.3. Superoxide Dismutase (SOD) Assay

The determination of total SOD enzyme activity (t-SOD) was performed using liver homogenate (80 µg) incubated with 880 µL of sodium carbonate (0.05%, pH 10.2, in EDTA 0.1 mM) in a water bath at 37 °C. Reaction was developed by addition of 20 µL of epinephrine (30 mM, in acetic acid 0.05%). SOD activity was measuring the kinetics of inhibition of epinephrine at 480 nm. One unit of SOD was defined as the amount of protein required to inhibit the autoxidation of 1 µmol de epinephrine per minute. Tissue t-SOD enzymatic activity was also expressed as units per mg of protein (U/mg protein) [30].

#### 3.7.4. Catalase (CAT) Assay

Liver homogenate (80 µg) was used to measure catalase activity. The principle of the assay is based on the determination of the rate constant *k* of H_2_O_2_ decomposition, which, in our conditions of temperature (30 °C) and pH (7.0), was defined as 4.6 × 10^7^. By measuring the absorbance changes (at 240 nm) per minute, for 2 min, the rate constant of the enzyme was determined. One unit of CAT was defined as the amount of protein required to convert 1 µmol de H_2_O_2_ per minute to H_2_O. Tissue CAT enzymatic activity was also expressed as mU/mg protein [31].

#### 3.7.5. Proliferating Cell Nuclear Antigen (PCNA) Imunohistochemical 

The PCNA analysis was performed in paraffinized rat liver. Slices (4 µm thick) were obtained and attached blade, deparaffinized in xylol and rehydrated. Antigen retrieval was performed using a microwave oven at 100 °C. After cooling at room temperature, endogenous peroxidase was blocked by peroxidase blocking solution. Sections were incubated with the primary antibodies for PCNA (anti-PCNA antibody [PC10]—proliferation marker (ab29)-mouse monoclonal antibody) (1:100), during 30 min, and then the sections were incubated with the secondary antibody using histofine Mouse Stain Kit (Nichirei Biosciences^®^, Tokyo, Japan) (1:200), during 30 min and washed in PBS. The reaction was visualized with diaminobenzidine (DAB), counter-stained with hematoxylin and mounted in resin. The PCNA reactivity analysis was performed by brown staining of the epithelial cells nuclei using the following scores: weak, moderate and intense reactivities.

### 3.8. Biochemical Parameters

The blood was collected was centrifuged at 1500× *g* for 10 min to obtain serum. Serum analysis of liver enzymes such as alanine aminotransferase (ALT), aspartate aminotransferase (AST), alkaline phosphatase (AF), γ-glutamyltranspeptidase (GGT), total bilirubin (TB), direct bilirubin (DB), indirect bilirubin (IB) and lactate dehydrogenase (LDH) and were estimated by using standard Boehringer Ingelheim^®^ diagnostic kits [32].

### 3.9. Statiscal Analysis

Statistical analysis was performed using Graph Pad Prism 5.0 software. The difference between groups was assessed by analysis of variance (ANOVA), followed, when necessary, by Newman-Keuls test. The significance level for rejection of the null hypothesis was always ≥5%.

## 4. Conclusions

SAAE has significant hepatoprotective effect on acute liver injury induced by CCl_4_. It might be postulated that its hepatoprotective action may be due to its inhibitory action on free radical formation by catechins as evident by the recovery of the antioxidant enzymes and by the decreased lipid peroxidation. The decreased biochemical parameters and PCNA immunoreactivity suggest that SAAE protects the hepatocyte against structural and functional damages by maintaining its integrity as well as increasing hepatocellular proliferation.
